# Supplementary material for: Activity Stabilization in a Population Model of Working Memory by Sinusoidal and Noisy Inputs
Source: Front Neural Circuits. 2021 Apr 21;15:647944. doi: 10.3389/fncir.2021.647944 (PMC8096914; doi:10.3389/fncir.2021.647944)
Supplement: Supplementary file 1 [file Data_Sheet_1.pdf]

Supplementary Material

1 Model

A generic multi-circuit model is described by the following equations:

$$\begin{cases}
\tau_{ra}\dfrac{dr_a^p}{dt} = -r_a^p + F_{ra}\left(\mu_a^p, \sigma_{a,AMPA}^p, \sigma_{a,GABAA}^p\right) \\
\mu_a^p = \mu_{a,AMPA}^p + \mu_{a,NMDA}^p + \mu_{a,GABAA}^p \\
\tau_{AMPA}\dfrac{d\mu_{a,AMPA}^p}{dt} = -\mu_{a,AMPA}^p + \sum_q \tilde{J}_{ae,AMPA}^{pq} K_{ae}\tau_{AMPA} r_e^q + \mu_{ax,BG}^p + \mu_{ax,NOISE}^p + \mu_{ax,STIM}^p + \mu_{ax,OSC}^p \\
\tau_{NMDA}\dfrac{d\mu_{a,NMDA}^p}{dt} = -\mu_{a,NMDA}^p + \sum_q \tilde{J}_{ae,NMDA}^{pq} K_{ae}\tau_{NMDA} r_e^q \\
\tau_{GABAA}\dfrac{d\mu_{a,GABAA}^p}{dt} = -\mu_{a,GABAA}^p + \sum_q \tilde{J}_{ai,GABAA}^{pq} K_{ai}\tau_{GABAA} r_i^q \\
\dfrac{\tau_{AMPA}}{2}\dfrac{d\left(\sigma_{a,AMPA}^p\right)^2}{dt} = -\left(\sigma_{a,AMPA}^p\right)^2 + \dfrac{1}{2}\sum_q \left(\tilde{J}_{ae,AMPA}^{pq}\right)^2 K_{ae}\tau_{AMPA} r_e^q + \left(\sigma_{ax,BG}^p\right)^2 \\
\sigma_{a,NMDA}^p = 0 \\
\dfrac{\tau_{GABAA}}{2}\dfrac{d\left(\sigma_{a,GABAA}^p\right)^2}{dt} = -\left(\sigma_{a,GABAA}^p\right)^2 + \dfrac{1}{2}\sum_q \left(\tilde{J}_{ai,GABAA}^{pq}\right)^2 K_{ai}\tau_{GABAA} r_i^q \\
\mu_{ax,NOISE}^p(t) = A_{ax,NOISE}^p \cdot \xi_{ax}^p(t) \\
\mu_{ax,STIM}^p(t) = A_{ax,STIM}^p \cdot \left[t_{STIM,1} \le t \le t_{STIM,2}\right] \\
\mu_{ax,OSC}^p(t) = A_{ax,OSC}^p \cdot \sin\left(2\pi f_{OSC}\cdot\left(t - t_{OSC,1}\right) + \varphi_{ax,OSC}^p\right)\cdot\left[t_{OSC,1} \le t \le t_{OSC,2}\right] \\
\dfrac{du^p}{dt} = -\dfrac{u^p}{\tau_F} + U(1-u^p)r_e^p \\
\dfrac{dx^p}{dt} = -\dfrac{(1-x^p)}{\tau_D} - u^p x^p r_e^p \\
\tilde{J}_{ab,S}^{pq} = \begin{bmatrix} J_{ab,S}^{pq} x^p u^p, \text{ if } p = q,\ ab = ee,\ S \in \left\{AMPA, NMDA\right\} \\ J_{ab,S}^{pq}, \text{ otherwise} \end{bmatrix}
\end{cases} \quad (1)$$

where the lower indexes *a*,*b* correspond to the population types (*e* – excitatory, *i* – inhibitory), whereas the upper indexes *p*,*q* are the number of a circuit. The variable r_a^p is the firing rate of a

population. The variable μ_a^p is the average input current, and $\mu_{a,AMPA}^p, \mu_{a,NMDA}^p, \mu_{a,GABAA}^p$ are the components of this current corresponding to the AMPA, NMDA ,and GABA synapses respectively; $\sigma_{a,AMPA}^p, \sigma_{a,NMDA}^p, \sigma_{a,GABAA}^p$ are the standard deviations of these current components (taken over the neurons of a population). The variables x^p, u^p describe the synaptic short-term plasticity. F_{ra} is the neuronal transfer function, τ_{ra} is the time constant of the population firing rate dynamics, and $\tau_{AMPA}, \tau_{NMDA}, \tau_{GABAA}$ are the synaptic time constants. K_{ab} is the number of synaptic projections from the population *b* to the population *a*. $J_{ae,AMPA}^{pq}, J_{ae,NMDA}^{pq}$ are the weights of the AMPA and NMDA connections from the excitatory population of the circuit q to the population a of the circuit p. $J_{ai,GABAA}^{pq}$ are the weights of the connections from the inhibitory population of the circuit q to the population a of the circuit p; $\tilde{J}_{ab,S}^{pq}$ $\left(S \in \{AMPA, NMDA, GABAA\}\right)$ are the weights of the connections with the short term plasticity taken into account. The variables $\mu_{ax,BG}^p$, $\sigma_{ax,BG}^p$ are the population average and standard deviation of the external background input from other brain areas that were not explicitly included into the model; $\mu_{ax,STIM}^p(t)$, $\mu_{ax,NOISE}^p(t)$, $\mu_{ax,OSC}^p(t)$ are the external input signals corresponding to the stimulus, the noise and the oscillations respectively. $A_{ax,STIM}^p$ is the stimulus amplitude, $t_{STIM,1}$ and $t_{STIM,2}$ are the start and end time of the stimulus; $A_{ax,NOISE}^p$ is the noise standard deviation, $\xi_{ax}^p(t)$ is the Gaussian white noise with the zero average and unit variance, $A_{ax,OSC}^p, f_{OSC}$ are the amplitude and frequency of the input oscillations, $t_{OSC,1}, t_{OSC,2}$ are the start and end time of the oscillatory input; τ_F, τ_D are the time constant of synaptic potentiation and depression respectively; U is the coefficient affecting the ratio between the potentiation and the depression.

For parameterization of the system, it is convenient to set the total level of excitation transmitted through the AMPA and NMDA receptors, as well as the ratio between the AMPA and NMDA components. Parameterization is also simplified by identity of the circuits. Thus, within-circuit connections could be described as follows:

$$\begin{cases} J_{ae,AMPA}^{pp} = J_{ae}(1-k_{NMDA}) \\ J_{ae,NMDA}^{pp} = J_{ae}k_{NMDA}\,\tau_{AMPA}/\tau_{NMDA} \\ J_{ai,GABAA}^{pp} = J_{ai} \end{cases}, \quad (2)$$

where J_{ae}, J_{ai} are the variables that characterize excitation and inhibition within the circuits; k_{NMDA} is a coefficient showing the contribution of the NMDA synapses in within-circuit excitation.

For the two-circuit model, we describe the inter-circuit weights as follows:

$$\begin{cases} J_{ee,AMPA}^{12} = J_{ee,AMPA}^{21} = J_{ee}^{cross}(1-k_{NMDA}^{cross}) \\ J_{ee,NMDA}^{12} = J_{ee,NMDA}^{21} = J_{ee}^{cross}(1-k_{NMDA}^{cross})\,\tau_{AMPA}/\tau_{NMDA} \\ J_{ab,S}^{12} = J_{ab,S}^{21} = 0, \quad ab \neq ee,\ S \in \{AMPA, NMDA, GABAA\} \end{cases}, \quad (3)$$

where J_{ee}^{cross} is the strength of the circuit interaction; k_{NMDA}^{cross} is a coefficient showing the contribution of NMDA current in circuit interaction.

Our multi-circuit model contains of two clusters. Each cluster contains two groups of 8 circuits each (which we denote as C1, I1 for the cluster 1 and C2, I2 for the cluster 2). The circuits from the first groups of the clusters (C1, C2) receive a common noise input, and the circuits from the second groups (I1, I2) – independent noise inputs.

For the multi-circuit model, we separately define the weights and NMDA coefficients for within-cluster and inter-cluster connections:

$$\begin{cases} \text{For } p,q \text{ from the same cluster:} \\ J_{ee,AMPA}^{pq} = c_{pq} J_{ee}^{clust}(1-k_{NMDA}^{clust}) \\ J_{ee,NMDA}^{pq} = c_{pq} J_{ee}^{clust}(1-k_{NMDA}^{clust})\,\tau_{AMPA}/\tau_{NMDA} \\ \text{For } p,q \text{ from different clusters:} \\ J_{ee,AMPA}^{pq} = c_{pq} J_{ee}^{far}(1-k_{NMDA}^{far}) \\ J_{ee,NMDA}^{pq} = c_{pq} J_{ee}^{far}(1-k_{NMDA}^{far})\,\tau_{AMPA}/\tau_{NMDA} \\ \text{For } all\ \ p \neq q: \\ J_{ab,S}^{pq} = 0, \quad ab \neq ee,\ S \in \{AMPA, NMDA, GABAA\} \end{cases}, \qquad (4)$$

where $J_{ee}^{clust}, J_{ee}^{far}$ are the strengths of within-cluster and inter-cluster connections, respectively; $k_{NMDA}^{clust}, k_{NMDA}^{far}$ are coefficients showing the contributions of the NMDA current in within-cluster and inter-cluster connections, respectively; c_{pq} is a binary coefficient showing presence or absence of a connection between circuits p and q. All the connections are symmetric: $c_{pq} = c_{qp}$.

The connectivity pattern $\{c_{pq}\}$ is chosen randomly according to the following rules:

- All the connections are symmetric: $c_{pq} = c_{qp}$;
- Each circuit within a group (C1, C2, I1, I2) receives 3 inputs from circuits of the same group;
- Different groups of the same cluster (C1-I1 and C2-I2) have 3 connections between each other, each circuit has either zero or one inter-group connection;
- Same groups of the different clusters (C1-C2, I1-I2) have 8 connections with each other, each circuit of a group has exactly one inter-cluster connection;
- Different groups of different clusters (C1-I2, C2-I1) are not connected.

Parameters of the tonic input, noise and external stimulus are the same for all circuits:

$$\begin{cases} \mu^{p}_{ax,BG} \equiv \mu_{ax,BG} \\ \sigma^{p}_{ax,BG} \equiv \sigma_{ax,BG} \\ A^{p}_{ax,NOISE} \equiv A_{ax,NOISE} \\ A^{p}_{ax,STIM} \equiv A_{ax,STIM} \end{cases} . \tag{5}$$

External oscillations are delivered to the excitatory populations of the circuits only. Amplitude of oscillations is the same for all circuits.

$$\begin{cases} A^{p}_{ex,OSC} \equiv A_{ex,OSC} \\ A^{p}_{ix,OSC} = 0 \end{cases} . \tag{6}$$

We consider either in-phase or anti-phase oscillatory inputs to circuits. In the two-circuit model, the circuits could receive oscillations in the same phase or in the opposite phases:

$$\begin{cases} \varphi^{1}_{ex,OSC} = \varphi^{1}_{ix,OSC} = \varphi^{2}_{ix,OSC} = 0 \\ \varphi^{2}_{ex,OSC} = \begin{bmatrix} 0, \text{ synphase} \\ \pi, \text{ antiphase} \end{bmatrix} \end{cases} \tag{7}$$

In the multi-circuit model, all circuits from a cluster receive an input of the same phase, while the phases could be the same or the opposite for the two clusters. In our notations:

$$\begin{cases} \varphi^{p}_{ix,OSC} = 0, \text{ for any } p \\ \varphi^{p}_{ex,OSC} = 0, \text{ for } p \text{ from the cluster 1} \\ \varphi^{p}_{ex,OSC} = \begin{bmatrix} 0, \text{ synphase} \\ \pi, \text{ antiphase} \end{bmatrix}, \text{ for } p \text{ from the cluster 2} \end{cases} \tag{8}$$

The noise input is delivered to the excitatory populations of the circuits only, with the same amplitude for all circuits:

$$\begin{cases} A^{p}_{ex,NOISE} \equiv A_{ex,NOISE} \\ A^{p}_{ix,NOISE} = 0 \end{cases} . \tag{9}$$

In the two-circuit model, the noise inputs to the circuits could be common ($\xi^{1}_{ex}(t) = \xi^{2}_{ex}(t)$) or independently generated. In the multi-circuit model, the noise input is identical for all the circuits that belong to the groups C1, C2 ($\xi^{p}_{ex}(t) = \xi^{COMMON}_{ex}(t)$), while each circuit from the groups I1, I2 receives independently generated noise.

The model parameters were set in such a way as to ensure metastability and resonant properties of the active regime. First, we selected parameters that ensure bistability in a single circuit (Fig. 2B).

In this case, a pair of complex-conjugate eigenvalues for the active state should correspond to slowly decaying oscillations in the gamma band (see Fig. S1B,D for example orbits). After that, the excitatory-to excitatory connection weight was decreased until the active state lost stability (Fig. 2A). Next, we considered two circuits and connected them with an excitatory connection, simultaneously decreasing the tonic inputs to the excitatory populations, in order to compensate for the inter-circuit excitation.

In the multi-circuit models, the inter-circuit weights were further decreased (compared to the two-circuit model), given the increased number of connections per circuit. The inter-cluster weights were made smaller that the within-cluster weights to keep the clusters relatively independent from each other. The proportion between the number of inter-group and within-group connections was made small enough, so the circuits receiving common noise could profit from it; otherwise, input correlations would spread across the whole system due to inter-circuit connectivity, compromising the difference between the "common-noise" and "independent-noise" groups. The noise intensity was set just enough for the activity duration difference between the "common-noise" and "independent-noise" groups to become visible. Finally, the oscillatory input amplitude was selected such that the oscillations considerably increase the activity duration and the difference between the "common-noise" and "independent-noise" groups both in the case of fast and slow inter-cluster connections.

The values of the model parameters are provided in Table 1.

Numerical simulations of the models were carried out using a self-developed software package in the Matlab environment. Numerical integration was performed using the Euler method with 0.2 ms time steps for the single-circuit and two-circuit models, and with 0.5 ms steps for the multi-circuit models.

Table 1. Model parameters

**Common parameters**			
**Parameter**	**Value**	**Parameter**	**Value**
τ_{re}	3 ms	K_e	400
τ_{ri}	1.5 ms	K_i	100
τ_e^{AMPA}	2 ms	J_{ee}	2.8 μA/cm^2
τ_e^{NMDA}	50 ms	J_{ie}	0.29 μA/cm^2
τ_i^{GABAA}	5 ms	k_{NMDA}	0.7
g_m	100 $\mu S/cm^2$	J_{ei}	-0.15 μA/cm^2
C_{me}	2 $\mu F/cm^2$	J_{ii}	-0.09 μA/cm^2
C_{mi}	1 $\mu F/cm^2$	$\mu_{ix,BG}$	0.25 μA/cm^2
E_L	-70 mV	$\sigma_{ex,BG}$	0.02 μA/cm^2

V_t	-50 mV	$\sigma_{ix,BG}$	0.02 μA/cm^2
V_r	-60 mV	τ_F	450 ms
U	0.03	τ_D	200 ms
**Single-circuit model**			
**Parameter**	**Value**	**Parameter**	**Value**
$\mu_{ex,BG}$	1 μA/cm^2	$A_{ex,NOISE}$	Control parameter
$A_{ex,STIM}$	5 μA/cm^2	f_{OSC}	Control parameter
$A_{ix,STIM}$	1 μA/cm^2	$A_{ex,OSC}$	Control parameter
$t_{STIM,1}$	200 ms	$\Delta\varphi_{OSC}$	0 or π
$t_{STIM,2}$	450 ms	$t_{OSC,1}$	800 ms
		$t_{OSC,2}$	Inf
**Two-circuit model**			
**Parameter**	**Value**	**Parameter**	**Value**
$\mu_{ex,BG}$	0.8 μA/cm^2	$A_{ex,NOISE}$	0.014 μA/cm^2
$A_{ex,STIM}$	5 μA/cm^2	f_{OSC}	40 Hz
$A_{ix,STIM}$	1 μA/cm^2	$A_{ex,OSC}$	Control parameter
$t_{STIM,1}$	200 ms	$\Delta\varphi_{OSC}$	0 or π
$t_{STIM,2}$	450 ms	$t_{OSC,1}$	800 ms
J_{ee}^{cross}	0.026 μA/cm^2	$t_{OSC,2}$	Inf
k_{NMDA}^{cross}	Control parameter		
**Multi-circuit model**			
**Parameter**	**Value**	**Parameter**	**Value**
$\mu_{ex,BG}$	0.8 μA/cm^2	$A_{ex,NOISE}$	0.02 μA/cm^2
$A_{ex,STIM}$	3.5 μA/cm^2	f_{OSC}	40 Hz
$A_{ix,STIM}$	0.7 μA/cm^2	$A_{ex,OSC}$	0.18 μA/cm^2

$t_{STIM,1}$	100 ms	ΔφOSC (within-cluster)	0
$t_{STIM,2}$	450 ms	$\Delta\varphi_{OSC}$ (inter-cluster)	0 or π
J_{ee}^{clust}	0.015 μA/cm^2	$t_{OSC,1}$	550 ms
k_{NMDA}^{clust}	0	$t_{OSC,2}$	Inf
J_{ee}^{far}	0.003 μA/cm^2		
k_{NMDA}^{far}	0.1 or 1		

2 Calculation of the transfer functions

The neuronal transfer functions relate the synaptic input to the value of the output firing rate. In our case, the arguments of the transfer function are the average synaptic current μ and the standard deviations of the excitatory AMPA-current σ_{AMPA} and the inhibitory GABAA-current σ_{GABAA}. To determine the value of the transfer function for a certain combination $(\mu, \sigma_{AMPA}, \sigma_{GABAA})$ we performed a simulation of a single leaky integrate-and-fire (LIF) neuron that receives a tonic current input μ and two noisy inputs with the zero average, standard deviations $\sigma_{AMPA}, \sigma_{GABAA}$, and time constants $\tau_{AMPA}, \tau_{GABAA}$:

$$\begin{cases} C_{ma} \dfrac{dV}{dt} = g_{La}(\mu - V) + I_{AMPA} + I_{GABAA} \\ if\ V = V_a^{TH} : V \leftarrow V_a^R \\ \tau_{AMPA} \dfrac{dI_{AMPA}}{dt} = -I_{AMPA} + \sigma_{AMPA}\sqrt{2\tau_{AMPA}}\eta_{AMPA}(t) \\ \tau_{GABAA} \dfrac{dI_{GABAA}}{dt} = -I_{GABAA} + \sigma_{GABAA}\sqrt{2\tau_{GABAA}}\eta_{GABAA}(t) \end{cases}, \qquad (10)$$

where C_{ma} is the membrane capacity, g_{La} is its conductivity, V_a^{TH} is a spike generation threshold, V_a^R is a reset voltage, I_{AMPA}, I_{GABAA} are the membrane currents through the AMPA- and GABAA-receptors respectively, and $\eta_{AMPA}(t)$, $\eta_{GABAA}(t)$ are independent implementations of Gaussian white noise with zero mean and unit variance. The parameter set is presented in the Table 2.

We carry out a simulation of the system (10) for different combinations $(\mu, \sigma_{AMPA}, \sigma_{GABAA})$, located in the nodes of a rectangular grid, and for each combination we stored the resulting firing rate. This procedure was performed twice: for an excitatory and for an inhibitory neuron. Thus, two three-dimensional arrays $\hat{F}_{re}, \hat{F}_{ri}$ were obtained. In the process of simulating the population model (1) and

analyzing the phase plane, we determined the values of the functions F_{re}, F_{ri} at the required points using cubic interpolation between the precomputed values $\hat{F}_{re}, \hat{F}_{ri}$ at the nearest grid nodes.

The shapes of the resulting transfer functions $F_{re}\left(\mu, \sigma_{AMPA}, \sigma_{GABAA}\right)$ and $F_{ri}\left(\mu, \sigma_{AMPA}, \sigma_{GABAA}\right)$ as functions of μ, given constant values of $\sigma_{AMPA}, \sigma_{GABAA}$, are presented in Figs. 2C,D, respectively. For this demonstration, we considered again a bistable single-circuit model (obtained by increasing J_{ee}). We plotted the shapes of F_{re} and F_{ri} as functions of μ for two different combinations of $\sigma_{AMPA}, \sigma_{GABAA}$ – one corresponding to the background state (solid curves in Figs. 2C,D) and the other – to the active state (dashed curves in Figs. 2C,D).

Table 2. The parameter set for computing of the transfer functions

**Parameter**	**Value**	**Parameter**	**Value**
C_{me}	2 µF/cm^2	$E_{Le} = E_{Li}$	-70 mV
C_{mi}	1 µF/cm^2	τ_{AMPA}	2 ms
$g_{Le} = g_{Li}$	100 µS/cm^2	τ_{NMDA}	50 ms
$V_e^{TH} = V_i^{TH}$	-50 mV	τ_{GABAA}	5 ms
$V_e^R = V_i^R$	-60 mV		

In our model, we assume that NMDA current variance is negligible and do not use it as an additional argument of the transfer functions. To justify it, we consider the following ratio:

$$\frac{\sigma_{NMDA}}{\sigma_{AMPA}} = \frac{J_{NMDA}\sqrt{K_e r_e \tau_{NMDA}}}{J_{AMPA}\sqrt{K_e r_e \tau_{AMPA}}} = \frac{J_{NMDA} K_e r_e \tau_{NMDA}}{J_{AMPA} K_e r_e \tau_{AMPA}} \sqrt{\frac{\tau_{AMPA}}{\tau_{NMDA}}} = \frac{\mu_{AMPA}}{\mu_{NMDA}} \sqrt{\frac{\tau_{AMPA}}{\tau_{NMDA}}} \,. \qquad (11)$$

If we assume that the mean currents are of the same order of magnitude ($\mu_{AMPA}/\mu_{NMDA} \sim 1$) and the NMDA time constant is much larger than the AMPA time constant ($\tau_{AMPA}/\tau_{NMDA} \approx 0$), then we conclude from (11) that $\sigma_{AMPA}/\sigma_{NMDA} \approx 0$.

3 Phase portrait analysis

For a deeper understanding of the system's behavior, we visualized a phase plane $\left(r_e, r_i\right)$ with the characteristic curves (Figs. 2A,B, S1). For each combination $\left(r_e, r_i\right)$, we determined the values of all other variables, at which their time derivatives turn to zero:

$$
\begin{cases}
u^*\left(r_e\right) = Ur_e\tau_F \Big/ \left(1 + Ur_e\tau_F\right) \\
x^*\left(r_e\right) = 1 \Big/ \left(1 + u^*\left(r_e\right) r_e \tau_D\right) \\
G_a^{syn}\left(r_e\right) = \begin{bmatrix} x^*\left(r_e\right) u^*\left(r_e\right), & a = e \\ 1, & a = i \end{bmatrix} \\
\mu_{a,AMPA}^*\left(r_e, r_i\right) = J_{ae,AMPA} G_a^{syn}\left(r_e\right) K_{ae} \tau_{AMPA} r_e + \mu_{ax,BG} \\
\mu_{a,NMDA}^*\left(r_e, r_i\right) = J_{ae,NMDA} G_a^{syn}\left(r_e\right) K_{ae} \tau_{NMDA} r_e \\
\mu_{a,GABAA}^*\left(r_e, r_i\right) = J_{ai,GABAA} K_{ai} \tau_{GABAA} r_i \\
\left(\sigma_{a,AMPA}^*\right)^2 \left(r_e, r_i\right) = \frac{1}{2} J_{ae,AMPA}^2 \left(G_a^{syn}\left(r_e\right)\right)^2 K_{ae} \tau_{AMPA} r_e + \sigma_{ax,BG}^2 \\
\left(\sigma_{a,GABAA}^*\right)^2 \left(r_e, r_i\right) = \frac{1}{2} J_{ai,GABAA}^2 K_{ai} \tau_{GABAA} r_i
\end{cases}
. \tag{12}
$$

We defined r_e -curve as the set of points on the plane $\left(r_e, r_i\right)$ for which the time derivative of r_e is zero, taking into account the conditions (12). Similarly, we defined r_i - curve as the set of points for which the time derivative of r_i is zero, and the conditions (12) are satisfied. Thus, we obtained the following expressions for both curves:

$$
\begin{cases}
r_e = F_{re}\left(\mu_e^*\left(r_e, r_i\right), \sigma_{e,AMPA}^*\left(r_e, r_i\right), \sigma_{e,GABAA}^*\left(r_e, r_i\right)\right) \\
r_i = F_{ri}\left(\mu_i^*\left(r_e, r_i\right), \sigma_{i,AMPA}^*\left(r_e, r_i\right), \sigma_{i,GABAA}^*\left(r_e, r_i\right)\right)
\end{cases}
. \tag{13}
$$

The intersections of the curves (two for a bistable system, one for metastable systems) correspond to the steady-states.

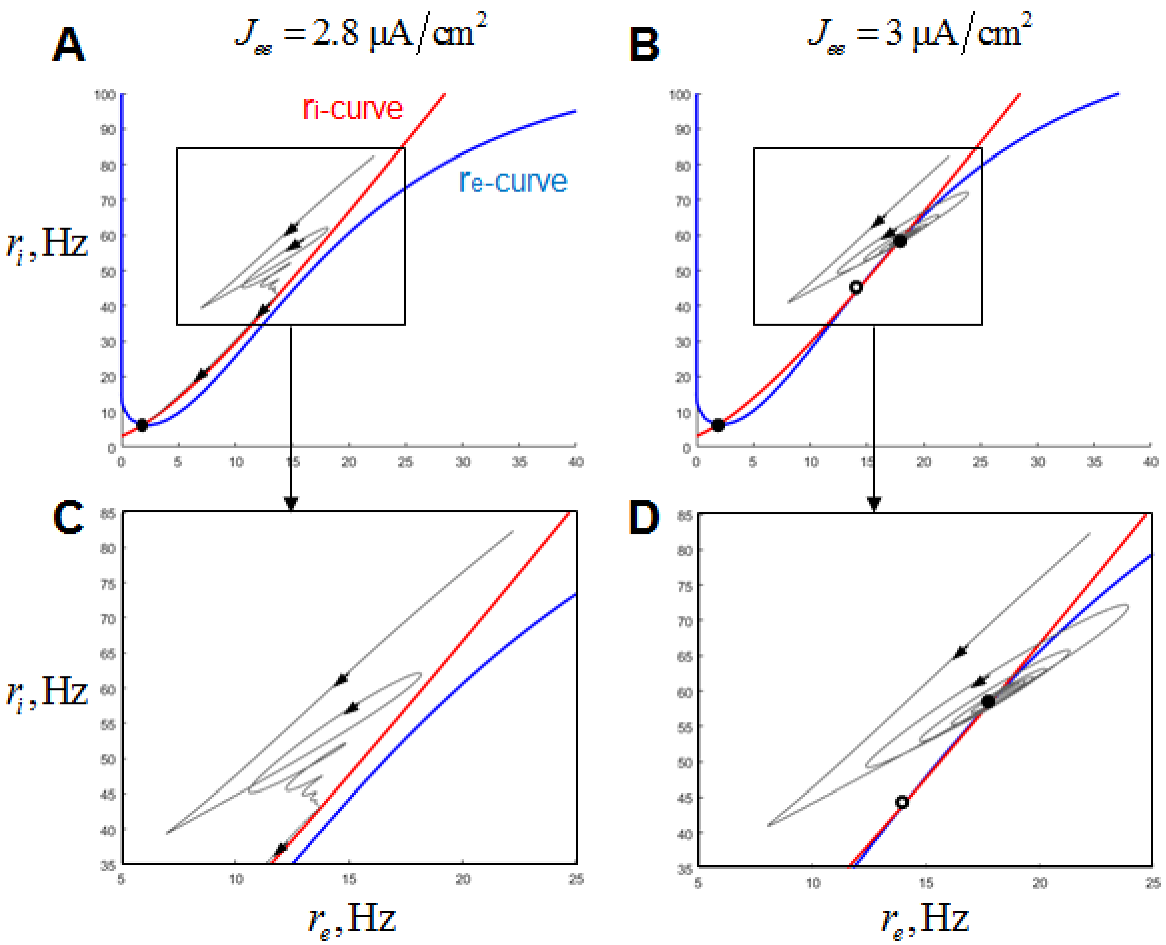

Figure S1. Damped oscillations on a phase plane. The legend is the same as in Fig. 2. (A, C) The system we analyzed in the paper, with the single steady-state (background) and the region of slowly decaying activity (where the re- and ri-curves are close to each other). The presented orbit approaches ri-curve in the region of high firing rates, demonstrating damped oscillations, and then goes towards the background steady state, staying close to ri-curve. (B, D) The system with increased recurrent excitation, with two steady-states (background and active). The presented orbit demonstrates damped oscillations around a point close to the active state and the converges to the active state itself. Since the presented phase plane is a projection of multi-dimensional state space, the orbits have self-intersections. For both systems, kNMDA was increased from the original value 0.7 to 0.73, for illustrative purposes (to increase the damping ratio).

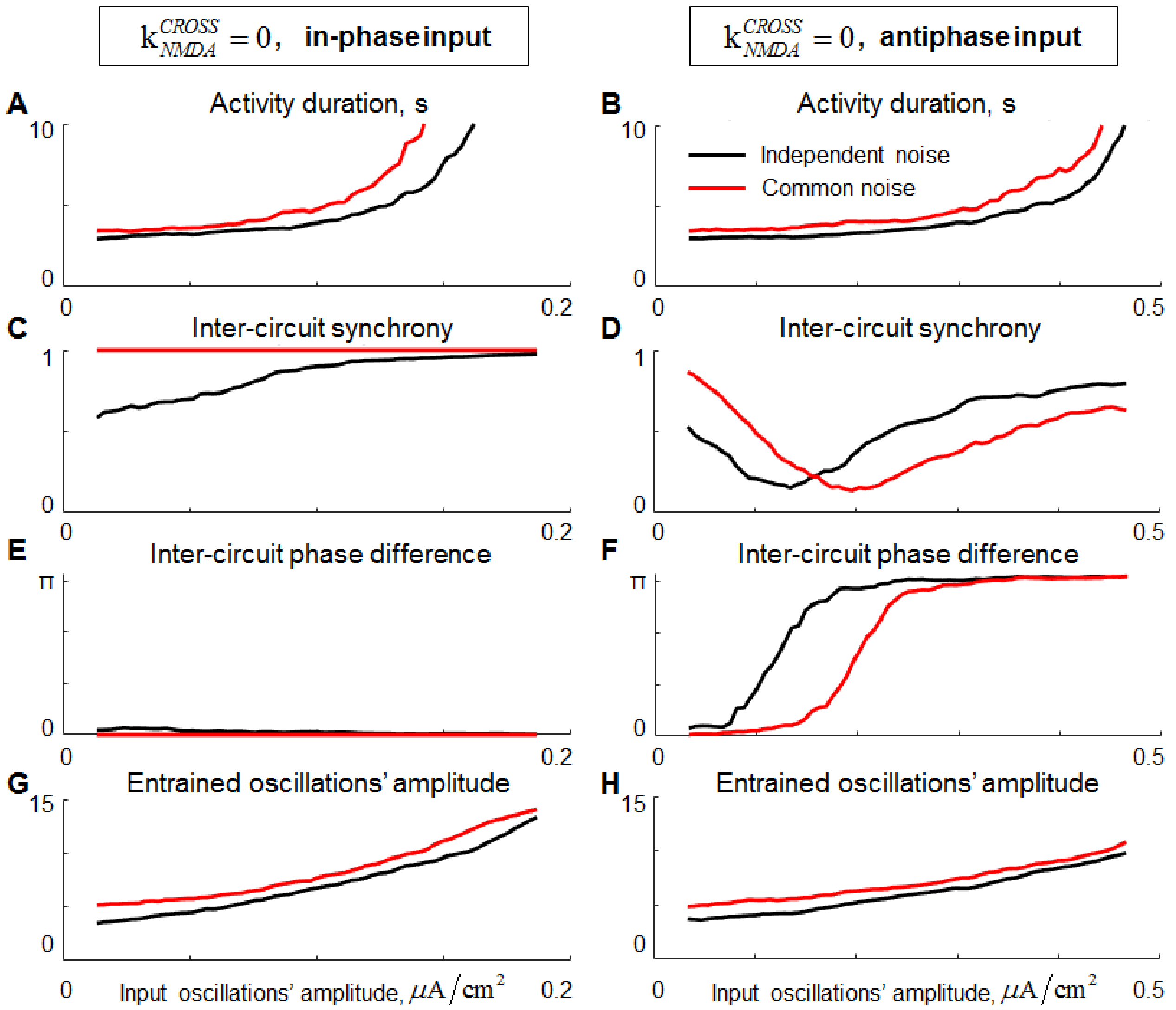

Figure S2. Behavior of the two-circuit model with fast inter-circuit connections under input oscillations of different amplitudes. Left column: the circuits receive oscillations in the same phase, right column – in opposite phases. Black curves: the circuits receive independent noise signals; red curves – a common noise signal. A, B – post-stimulus activity duration, averaged over the two circuits. The activity of a circuit is considered to be terminated when the firing rate of the excitatory population, smoothed with 100-ms time window, falls below 3 Hz. C, D – inter-circuit synchrony measured as the phase-locking value between the excitatory firing rate signals. E, F – Mean phase difference between the excitatory firing rate signals. G, H – Mean amplitude of the entrained oscillations of the excitatory firing rate signal. For calculation of C – H, the firing rate dynamics of the excitatory population of each circuit was bandpass-filtered (30 – 50 Hz), after which the Hilbert transform was applied to extract the amplitude and phase of the filtered signal at each time moment.

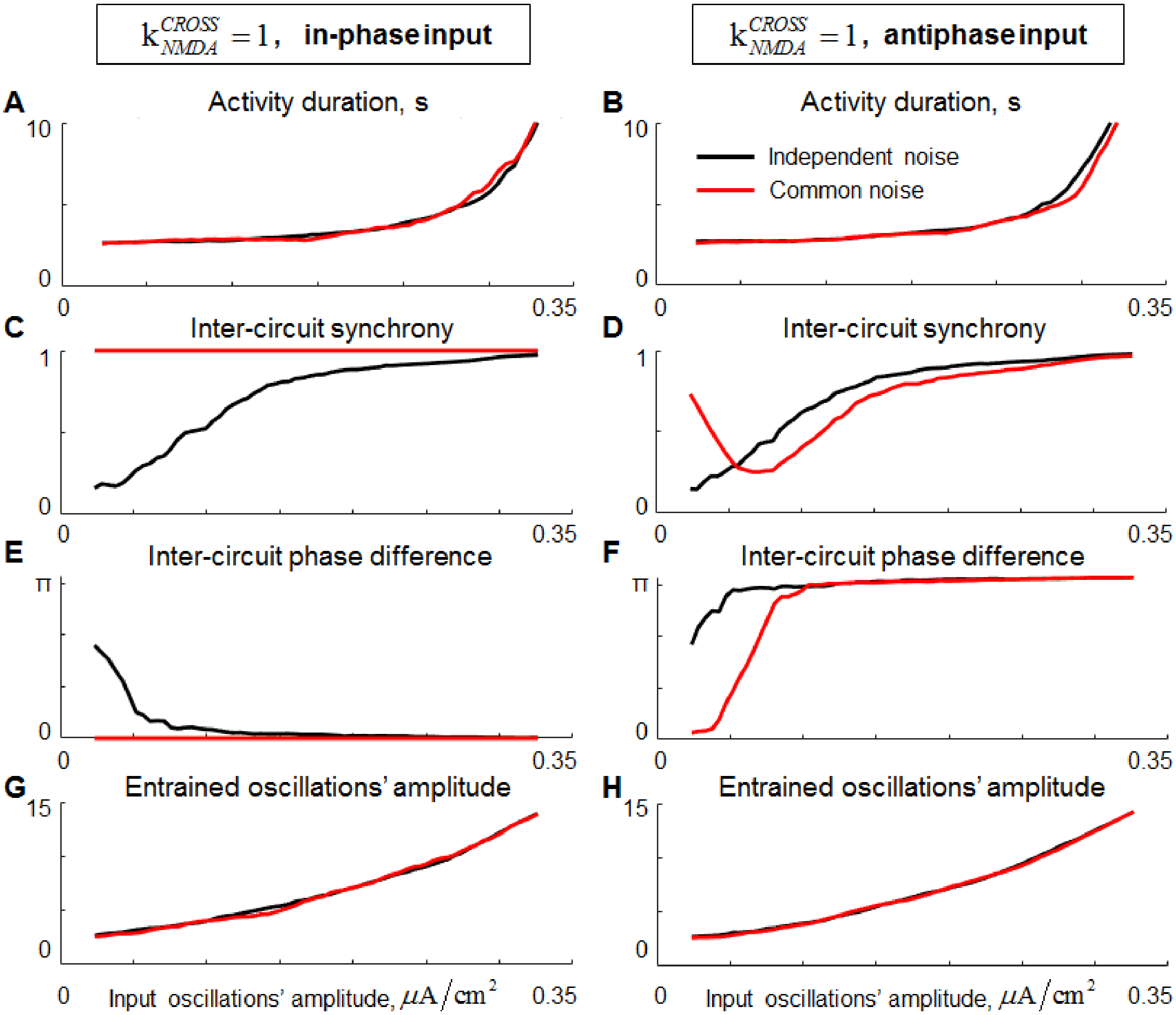

Figure S3. Behavior of the two-circuit model with slow inter-circuit connections under input oscillations of different amplitudes. The legend is the same as in Fig. S2.
